# Laparoscopic Video Analysis Using Temporal, Attention, and Multi-Feature Fusion Based-Approaches

**DOI:** 10.3390/s23041958

**Published:** 2023-02-09

**Authors:** Nour Aldeen Jalal, Tamer Abdulbaki Alshirbaji, Paul David Docherty, Herag Arabian, Bernhard Laufer, Sabine Krueger-Ziolek, Thomas Neumuth, Knut Moeller

**Affiliations:** 1 Institute of Technical Medicine (ITeM), Furtwangen University, 78054 Villingen-Schwenningen, Germany; 2 Innovation Center Computer Assisted Surgery (ICCAS), University of Leipzig, 04103 Leipzig, Germany; 3 Department of Mechanical Engineering, University of Canterbury, Christchurch 8041, New Zealand; 4 Department of Microsystems Engineering, University of Freiburg, 79110 Freiburg, Germany

**Keywords:** context-aware system, laparoscopic video analysis, surgical phase recognition, surgical tool classification, surgical tool localization

## Abstract

Adapting intelligent context-aware systems (CAS) to future operating rooms (OR) aims to improve situational awareness and provide surgical decision support systems to medical teams. CAS analyzes data streams from available devices during surgery and communicates real-time knowledge to clinicians. Indeed, recent advances in computer vision and machine learning, particularly deep learning, paved the way for extensive research to develop CAS. In this work, a deep learning approach for analyzing laparoscopic videos for surgical phase recognition, tool classification, and weakly-supervised tool localization in laparoscopic videos was proposed. The ResNet-50 convolutional neural network (CNN) architecture was adapted by adding attention modules and fusing features from multiple stages to generate better-focused, generalized, and well-representative features. Then, a multi-map convolutional layer followed by tool-wise and spatial pooling operations was utilized to perform tool localization and generate tool presence confidences. Finally, the long short-term memory (LSTM) network was employed to model temporal information and perform tool classification and phase recognition. The proposed approach was evaluated on the Cholec80 dataset. The experimental results (i.e., 88.5% and 89.0% mean precision and recall for phase recognition, respectively, 95.6% mean average precision for tool presence detection, and a 70.1% F1-score for tool localization) demonstrated the ability of the model to learn discriminative features for all tasks. The performances revealed the importance of integrating attention modules and multi-stage feature fusion for more robust and precise detection of surgical phases and tools.

## 1. Introduction

Recent innovations in the medical field have led to the proliferation of technological advances inside operating rooms (ORs). As advanced as today’s operating theaters are, increasing surgical workflow complexity, the emergence of new needs of clinicians, and new patient preferences, from one side, and advances in data science, artificial intelligence (especially deep learning), and computer vision, from the other side, are all likely to be key features in future ORs [1,2,3]. By integrating intelligent context-aware systems (CASs), future ORs will enable the transform from clinicians’ knowledge-based to a more data-driven surgical treatment. Indeed, data-driven treatments include perceptively interacting with medical teams (e.g., surgical and anesthesiological teams), enabling multi-perspective knowledge-sharing between medical teams, providing medical support, and mitigating possible complications [2,4]. In this context, CAS should be able to conceive the workflow inside the OR, understand the current situation by fusing data from different perspectives (surgical and patient-related data) [5,6], and predict upcoming surgical events. Thus, analyzing surgical workflow inside the OR represents a central goal of CAS [7,8].

Analyzing a surgical workflow relies on modeling surgical procedures as surgical process models (SPMs) [8]. In this context, surgical workflows can be described as sequences of surgical phases that represent the main tasks performed during surgery [7,8]. Surgical phases consist of goal-specific high-level tasks. Hence, different granularity levels have been defined to model the surgical procedure [8]. In fact, recognizing surgical phases and detecting surgical tools have great potential in providing intra-operative and post-operative assistance for clinicians. Recognizing the current surgical phase and predicting upcoming phases help in promoting better situational awareness inside the OR and providing medical support to the surgical team by detecting abnormal cases. Moreover, the duration of the surgical procedure can be estimated and the schedule of the surgical department as well as resources can be optimized [9]. Automatic surgical phase and tool recognition systems can also be utilized to label recorded data and, therefore, provide trainees with training materials.

In the domains of surgical phase recognition and tool detection, the described approaches relied on different data sources, such as surgical videos (laparoscopic [10], microscopic videos [11]), sensor data [12,13], instrument sensors [14,15], and medical device data [16]. As laparoscopic surgery became an established surgical practice and nearly replaced open surgery, significant research was conducted on laparoscopic video data. The main advantages of laparoscopic videos over other data sources are that they are already integrated into the current setup inside ORs, can be easily accessed and captured, and can provide comprehensive information about the surgical instruments used, the anatomies treated, and activities conducted. On the other hand, analyzing laparoscopic videos has been a challenging task for researchers in the field of surgical data science (SDS) [2]. Extensive efforts have been made to develop video-based approaches for the automatic recognition of surgical phases [17] and the detection and localization of surgical tools. Earlier approaches relied on extracting visual features from laparoscopic images and then employing an adequate classifier [10,18,19]. In recent years, the present-day evolution in deep learning (DL), triggered by the development of high-performance hardware infrastructure, has shifted the focus to DL-based approaches rather than relying on traditional machine learning approaches. Interestingly, DL techniques, specifically convolutional neural networks (CNNs), have shown superior performance to other methods [20].

In this work, a spatiotemporal, a weakly-supervised deep learning approach for analyzing laparoscopic surgical videos (in terms of surgical phase recognition, surgical tool classification, and localization) was proposed. Initially, ResNet-50 was chosen as the base model, but with the following modifications: First, four squeeze-and-excitation (SE) attention modules were adopted to the CNN architecture to enhance the CNN capability to learn more discriminative features and focus on tool-related regions in the image; Second, feature maps from low and top layers were aggregated to generate a better representation of image content. The aggregated features were then shared by two branches (tool and phase branches). Following a similar trend as the earlier approaches [21], the tool branch contained a convolutional layer to generate multiple feature maps per tool class. By implementing tool-wise pooling and spatial pooling operations, the tool-related feature maps were transferred into a localization map and tool-presence confidence, respectively. The phase branch was composed of a global average pooling (GAP) layer followed by a concatenating layer to include tool presence probabilities in the final feature vector for phase recognition. The LSTM network was finally employed to model temporal information that is crucial for phase recognition and tool presence detection tasks. The proposed model was evaluated on the Cholec80 dataset [22].

## 2. State of the Art

For surgical phase recognition, several DL approaches are presented in the literature, including spatial and temporal models. Indeed, temporal information along the surgical video sequence is essential to model dependencies between surgical phases that are typically performed in a specific order [17]. Therefore, a base CNN model, such as ResNet-50 [23] or VGG-16 [24], was first adapted and utilized for extracting spatial features from laparoscopic images. Then, a temporal model (such as the hidden Markov model (HMM) [22,25] or recurrent neural network (RNN) [26,27,28]) was incorporated to refine the CNN predictions. Twinanda et al. presented a multi-task CNN model that performed surgical phase recognition and tool classification [22]. Hierarchical HMM (HHMM) was employed as a temporal model to perform online and offline recognition of surgical phases. To overcome drawbacks imposed by statistical models, later approaches implemented long short-term memory (LSTM) networks to learn temporal features [26,28,29]. For example, Twinanda et al. substituted the HHMM models in the EndoNet methodology with an LSTM model [26]. Jin et al. proposed a CNN-LSTM deep learning framework (SV-RCNet) trained end-to-end with a prior knowledge inference scheme to carry out phase recognition [29]. Similarly, Jin et al. devised the MTRCNet approach that performed both the surgical phase and tool recognition and employed a novel loss function that considered the phase-tool relation [27]. Jalal et al. suggested using a nonlinear autoregressive network with exogenous input (NARX) with a CNN for surgical phase prediction [28]. In [30], a temporal approach called TeCNO, which combined a ResNet-50 model with a multi-stage temporal convolutional network (MS-TCN), was proposed. Recently, various transformer-based models tailored for laparoscopic phase recognition have been introduced [31]. For instance, Czempiel et al. designed the OperA approach that is based on a transformer model to concurrently learn spatial and temporal features along video sequences [32]. Gao et al. employed a hybrid embedding aggregation transformer to aggregate spatial and temporal features generated by ResNet-50 and TCN models [33].

Surgical tool classification and localization were tackled in a similar fashion to phase recognition, and several methods suggested multi-task models for surgical presence detection and phase recognition. In [22], surgical tool classification was conducted solely based on spatial features learned by a CNN model. Subsequent studies addressed typical challenges facing tool classification methods, such as imbalanced data distribution and obscured images. Loss-sensitive and resampling techniques were introduced in [34] to mitigate the effects of the imbalanced distribution of surgical tools on the CNN training process. Spatiotemporal models were introduced to refine tool predictions obtained by the CNN model. Several temporal models, such as LSTM [35,36,37], graph convolutional networks (GCN) [38], and convolutional LSTM [39], were presented in previous works. Abdulbaki Alshirbaji et al. proposed combining a CNN model with two-stage LSTM models to model temporal dependencies in short video clips and along the complete surgical video sequence [35]. Despite the various successful approaches that have been developed, the progress achieved in the SDS field is still limited and still lacks significant applications in practice [2]. The main reason is the scarcity of labeled surgical data. Therefore, several techniques have been introduced to increase the size of data used for training CNN models [40]. These include data augmentation and generative adversarial network (GAN) [41]. Moreover, weakly-supervised learning of CNN models represents a potential solution for object localization. Here, the CNN is designed to perform object localization but trained only with object presence binary labels. Durand et al. suggested an approach for weakly-supervised object localization by adding a multi-map localization layer on top of the CNN model [21]. They also introduced a novel spatial pooling strategy to transform the multi-maps into a class-wise localization map. Their approach was investigated by Vardazaryan et al. [42] and Nwoye et al. [39] for surgical tool localization in laparoscopic videos, and it performed very well.

Recently, CNN-attention networks have been proposed; they involve adopting attention modules into the CNN architecture to help generate more focused features. Shi et al. proposed an attention-based CNN to perform surgical tool detection in laparoscopic images [43]. In [44], an attention-guided network (AGNet) for surgical tool presence detection achieved high performance on the m2cai16-tool dataset. Furthermore, Jalal et al. emphasized the value of employing attention modules for surgical tool localization in their feasibility study [45]. Attention CNN was capable of generating more fine and focused gradient class activation maps (Grad-CAM) that were utilized to extract bounding boxes. The proposed approaches were evaluated on a single dataset or a single type of surgical procedure. Therefore, the robustness and generalizability of deep learning approaches toward new data represent the main concerns that need to be investigated before translating these approaches into clinical practice [46].

## 3. Materials and Methods

### 3.1. System Architecture

**Figure 1:**
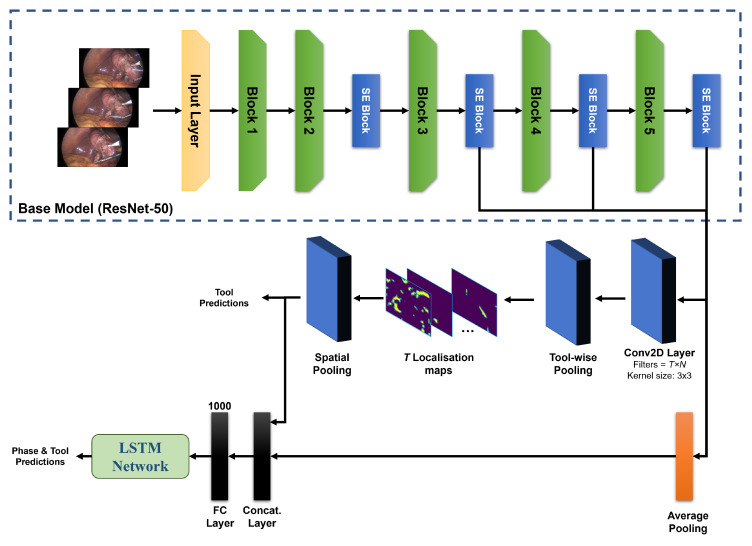
An overview of the proposed approach for phase recognition, tool localization, and classification. SE blocks are the squeeze-and-excitation attention modules; the Conv2D layer represents the multi-map convolutional layer where *T* is the number of tool classes in the dataset, and *N* is the number of feature maps generated per tool.

The proposed architecture for laparoscopic surgical video analysis (presented in Figure 1) consists of ResNet-50 as a backbone model followed by two branches for phase recognition, tool localization, and classification. The ResNet-50 model was modified by incorporating four SE-attention blocks and adding multi-stage feature fusion connections. The tool localization branch is composed of a multi-map convolutional layer for tool localization, as well as tool-wise and spatial pooling layers. The tool-wise and spatial pooling outputs represent the tool localization maps and tool presence confidence, respectively. The phase recognition branch consists of a GAP layer to transfer the feature maps from the last SE module into a feature vector, a concatenating layer that combines the feature vector from the GAP layer with tool presence probabilities obtained from the spatial pooling layer, a fully-connected layer (FC), and finally an LSTM layer to model temporal dependencies along the laparoscopic video.

#### 3.1.1. Backbone CNN Model

Based on previous work [35], the ResNet-50 performed better than other base CNN models for surgical tool classification. Therefore, the ResNet-50 was chosen to perform this study. The ResNet-50 is composed of 5 blocks with output feature maps of 64, 256, 512, 1024, and 2048, respectively. The first block consists of 1 convolutional layer followed by a max pooling operation, while the other four blocks are built as a stack of 3, 4, 6, and 3 residual units. Each residual unit consists of a stack of three convolutional layers, each followed by a batch normalization and a rectified linear unit (ReLU) layer. The network has (on top) a GAP layer, an FC layer, and softmax. The input image size of the ResNet-50 is 224 × 224 × 3. To maintain spatial information, the following modifications were introduced: first, the spatial dimension of the input was increased to 375 × 300; second, the strides of the convolutional layers were set to 1 × 1 (similar to Vardazaryan et al. [42]) to preserve the higher resolutions of obtained feature maps.

#### 3.1.2. Squeeze-and-Excitation (SE) Attention Modules

**Figure 2:**
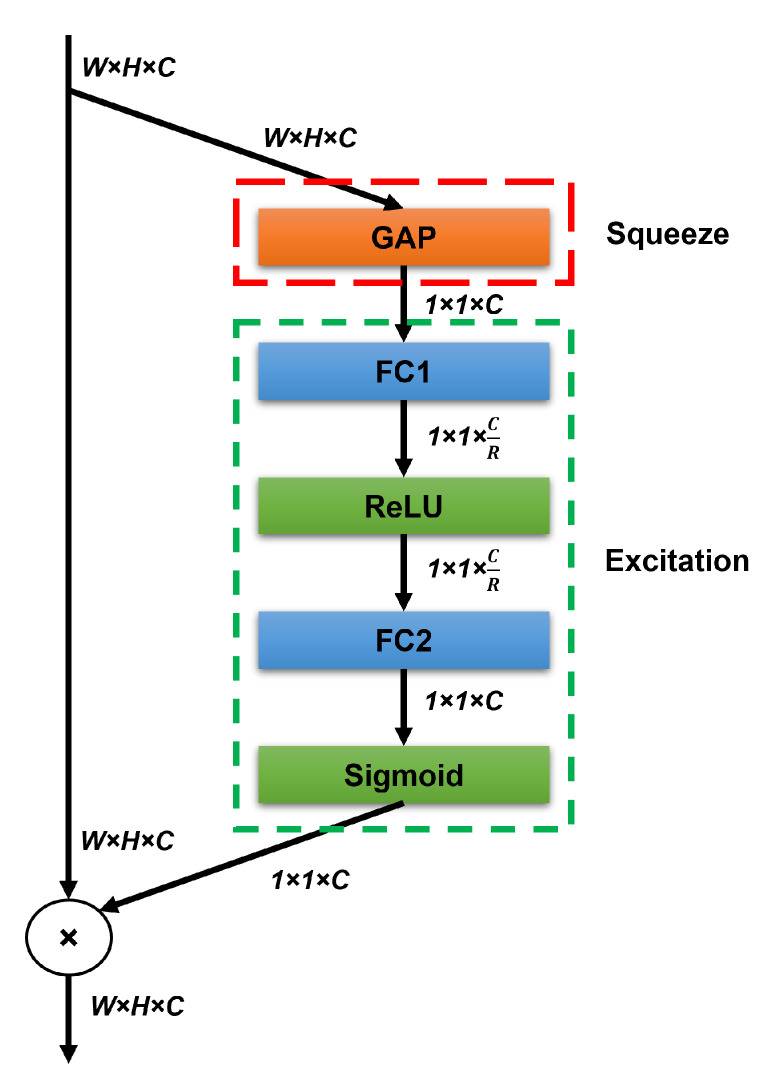
Squeeze-and-excitation attention module architecture.

Attention modules were adapted to the ResNet-50 model to enhance the ability of the CNN to learn more focused and important information related to the surgical tools rather than the background information. Squeeze-and-excitation (SE) attention module [47] was chosen in this study due to its efficient and fast computational performance and its potential to improve performance over base CNN models [48]. The SE considers the relationship between feature channels and recalibrates the channel-wise features through the squeeze and excitation operations (Figure 2). The squeeze operation compresses the input W×H×C feature maps into a 1 × 1 × C vector by using GAP (Equation (1)). The excitation operation learns weights for each feature channel to model the dependencies between feature channels (Equation (2)). Two FC layers are employed to perform the excitation operation, where the first FC reduces the dimensionality by a reduction factor *R*, and the second FC expands the input data back to the original dimension. The two FC layers are followed by ReLU and Sigmoid activation layers, respectively. The output of the Sigmoid is then multiplied by the input feature maps of the squeeze operation generating more focused features. The squeeze and excitation operations are represented as

(1) $$Z_{n}=\sum_{i=1}^{W}\sum_{j=1}^{H}y_{n}\left(i,j\right)$$

where $y_{n}\in R^{W\times H}$ represents a feature map, *H* and *W* are the feature map height and width, $Z_{n}$ is an element of $Z\in R^{C}$.

(2) $$E=Sigmoid(W_{2}\times ReLU\left(W_{1}Z\right))$$

where $W_{1}\in R^{\frac{C}{R}\times R}$ and $W_{2}\in R^{C\times \frac{R}{C}}$ are the weights of the first and second FC layers, respectively, and *R* is the reduction factor.

Four SE modules were used in this study and added after the second, third, fourth, and fifth convolutional blocks of the ResNet-50 (see Figure 1). The number and locations of added SE modules were specified based on an extensive evaluation carried out and previously published in [48]. The *R* was selected to be 16 for all SE blocks.

#### 3.1.3. Multi-Stage Feature Fusion (MSF)

Traditional CNN models rely on providing the features from the top layer to an FC layer to perform classification. Recently, the aggregation of low-level and high-level features showed improvement in object classification accuracy over using only high-level features [49]. Additionally, while high-level features from top convolutional layers contain semantic information for target classes, features from shallow convolutional layers represent generic features describing detailed information at the instance level. Therefore, fusing features obtained at different levels of the CNN model has the potential to enhance the generalization capability of the CNN and provide a better representation of the input space [50].

In this study, feature maps from three intermediate layers were combined with the feature maps of the last layer and utilized later to perform phase recognition, tool classification, and localization. The outputs of the second, third, and fourth SE blocks were first passed through a batch normalization (BN) and a regularization (ReLU) layer. The outputs from the ReLU layers were then concatenated and forwarded to the multi-map convolutional layer and a GAP layer (Figure 1).

#### 3.1.4. Multi-Map Convolutional Layer (MMC)

To obtain localization feature maps of each tool, a convolutional layer, termed multi-map convolutional layer was added to learn tool-related spatial features. The convolutional layer has T×N filters and a kernel size of 3×3. *T* is the number of tool classes (T=7 for Cholec80 dataset), and *N* represents the number of feature maps for each class (N=4 was chosen for this study). The stride was set to 1×1 to maintain high spatial resolution. The output dimensionality of the MMC layer is $W_{2}\times H_{2}\times TN$. Therefore, the output of this convolutional layer consists of four feature maps per tool class. These feature maps were learned by the training the model with binary tool presence labels. The MMC layer had a Sigmoid activation function.

#### 3.1.5. Tool-Wise and Spatial Pooling Layers

The four feature maps of each tool were transferred into a localization map by applying tool-wise pooling. Max pooling operation was applied across the *M* feature maps. Therefore, the tool-wise pooling transferred the feature maps from $W_{2}\times H_{2}\times TN$ to $W_{2}\times H_{2}\times T$. These maps were utilized to obtain localization maps of the surgical tools and allocated bounding boxes.

To obtain estimation confidence of the tools, the output of the tool-wise pooling was then passed through spatial pooling. The spatial pooling introduced by Durand et al. [21] was implemented in this study. Giving M the output of the class-wise pooling, ${\tilde{M}}_{max}$ the top maximum $K_{max}$ elements of M, and ${\tilde{M}}_{min}$ the lowest minimum $K_{min}$ of M, the spatial pooling equation can be described as

(3) $$S=\frac{1}{K_{max}}\sum_{i,j}{\tilde{M}}_{max}+\alpha \left(\frac{1}{K_{min}}\sum_{i,j}{\tilde{M}}_{min}\right)$$

where $K_{max}$ and $K_{min}$ are chosen to be equal to 50.

#### 3.1.6. LSTM Network

The recognition of surgical phases and detection of tools require learning static and sequential information along the laparoscopic video. Therefore, an LSTM network was employed to learn temporal features. The LSTM network has a sequence-to-sequence configuration and was trained with sequences of spatial feature vectors. Every feature vector represents the features of each image extracted using the GAP layer combined with tool presence confidences. An LSTM network of one LSTM layer with 512 cells was chosen for this study.

### 3.2. Model Evaluation

#### 3.2.1. Dataset

The Cholec80 dataset [22] was used in this study for evaluating the performance of the proposed approach. The Cholec80 is composed of 80 videos of cholecystectomy procedures labeled with surgical phases and presented surgical tools. Seven surgical phases and seven tools have been defined. Details about the surgical phases and tools are presented in Table 1. The videos were recorded at 25 frames-per-second (fps), but the tool labeling was carried out at 1 fps. The tool was defined as present if at least half of its tip was visible. In accordance with previous studies, the first 40 videos of the Cholec80 dataset were used for training the model, while the last 40 videos were used for model evaluation.

Since surgical tools were labeled with only binary presence signals, another small dataset (termed Cholec80-Boxes) was created for the evaluation of the tool localization task. Here, the first five videos of the test set were labeled with bounding boxes around the tool`s characteristic tip. The bounding box labeling was carried out by two medical engineers using the MATLAB Video Labeler toolbox (R2021a, The MathWorks, Natick, MA, USA).

#### 3.2.2. Evaluation Criteria

The mean average precision (mAP) was utilized as an evaluation metric for surgical tool presence detection. The AP was first calculated for each tool by computing the area under the precision–recall curve, and the mAP was then obtained by calculating the average overall tool classes.

For tool localization, the F1-score metric was utilized and computed as in Equation (4). First, the intersection over union (IoU) between the predicted bounding box and the manually labeled bounding box was calculated. The predicted bounding box was counted as true positive prediction if the tool presence confidence and the IoU exceeded certain thresholds $T_{C}=0.5$ and $T_{IoU}=0.5$, respectively. If the predicted bounding box had an IoU and tool confidence lower than the $T_{IoU}$ and $T_{C}$, respectively, it was counted as a false positive prediction. False negative predictions represented bounding boxes with tool confidences lower and greater than $T_{C}$ but with IoU lower than $T_{IoU}$.

(4) $$\begin{array}{c} F1=2\frac{Precision*Recall}{Precision+Recall} \end{array}$$

To evaluate the performance of surgical phase recognition, the precision and recall were utilized and computed for each phase as

(5) $$Precision=\frac{Ph_{G}\cap Ph_{P}}{Ph_{P}},Recall=\frac{Ph_{G}\cap Ph_{P}}{Ph_{G}}$$

where $Ph_{G}$ is the phase ground truth and $Ph_{P}$ is the phase prediction.

#### 3.2.3. Training Setup

For the tool presence detection task, three approaches were compared in this work. The first approach is termed ***CNN-MMC*** and is composed of the ResNet-50 model combined with the MMC and the tool-wise and spatial pooling layers. The second approach is termed ***CNN-SE-MSF*** and consists of the ***CNN-MMC*** but adapted by adding SE attention modules and the MSF. The third approach is composed of the ***CNN-SE-MSF*** combined with an LSTM network to model temporal dependencies along the surgical video and is termed ***CNN-SE-MSF-LSTM***. For the tool localization task, the ***CNN-MMC*** and ***CNN-SE-MSF*** approaches were compared. Table 2 presents a summary of the applied approaches.

The ImageNet weights of the ResNet-50 transferred layers were utilized as initial values, while added layers were initialized with random weights. The losses for tool classification and phase recognition tasks were computed using binary cross-entropy (Equation (6)) and softmax multinomial logistic (Equation (7)) functions, respectively. To compensate for the effects of the imbalanced distribution of the tools, loss-sensitive learning was applied by weighing the loss of each tool based on its distribution in the training set.

(6) $$Tool_{loss}=\frac{-1}{B}\sum_{n=1}^{B}\sum_{t=1}^{T}w_{t}\left[l_{t}^{n}\log\left(C_{t}^{n}\right)+\left(1-l_{t}^{n}\right)\log(1-C_{t}^{n}\right)]$$

where $Tool_{loss}$ is the total loss of all tools, *B* is the batch size, *T* is the number of tools in the dataset, $w_{t}$ represents the loss weight calculated for every surgical tool, $l_{t}^{n}=\left[0,1\right]$ is the tool presence ground truth, and $C_{t}^{n}$ is the tool presence confidence obtained from the spatial pooling operation.

(7) $$Phase_{loss}=\frac{-1}{B}\sum_{n=1}^{B}\sum_{p=1}^{P}G_{p}^{n}\log\sigma (P_{p}^{n})$$

where $Phase_{loss}$ is the total loss of surgical phases, *B* is the batch size, *P* is the number of defined phases in the dataset, $G_{p}^{n}$ is the ground truth of image *n*, $P_{p}^{n}$ is the output of the FC layer and σ represents the softmax activation function.

An Adam optimizer and cyclical learning rate [51] were implemented to eliminate the need to find the best values for the learning rate. The bounds of the cyclical learning rate were 0.005 and 0.000001, and a step size of 4×iteration−per−epoch was chosen. The CNN model was trained with a batch size of 50 images, and the images were shuffled for every epoch. All spatial models were trained for 30 Epochs. The LSTM was trained with complete video sequences with a batch size of 1 video for 50 Epochs. The implementation of the models was performed in the Keras framework with the Anaconda platform and run on an NVIDIA RTX A6000 graphics processing unit (GPU).

## 4. Results

**Figure 3:**
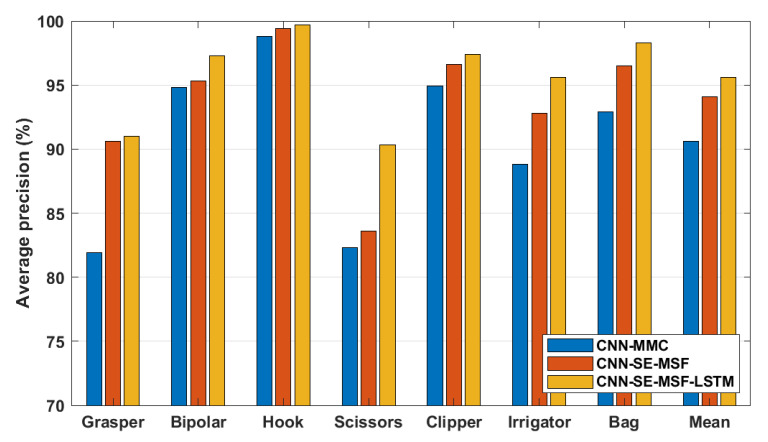
Tool presence average precision (AP) on the Cholec80 dataset. Note the truncated scale of the y-axis.

**Figure 4:**
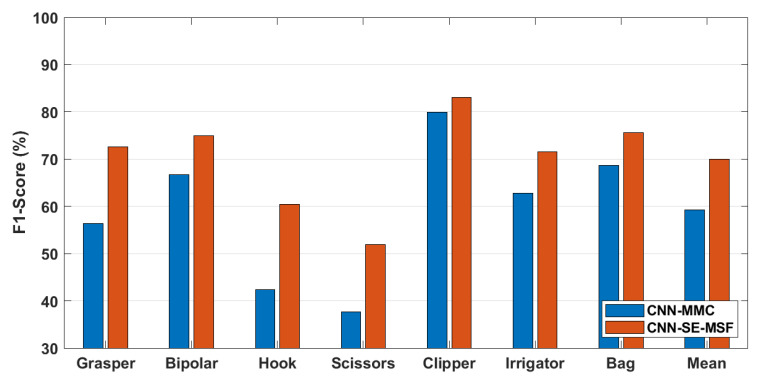
Tool localization F1-score on the Cholec80-Boxes dataset. Note the truncated scale of the y-axis.

The results of the tool presence detection obtained by the ***CNN-MMC***, ***CNN-SE-MSF***, and ***CNN-SE-MSF-LSTM*** approaches are shown in Figure 3. Figure 4 shows the F1-score for tool localization using the ***CNN-MMC*** and ***CNN-SE-MSF*** approaches. From both figures, the results show the value of adding the attention modules and combining features from multiple stages to improve tool presence detection and generate better localization maps for all tools. The average precision of all tools was enhanced by a large margin over the ***CNN-MMC***, and the most notable enhancement was achieved after employing the LSTM network. To further validate the results of the proposed approach, tool-wise comparisons between the ***CNN-SE-MSF-LSTM*** and the state-of-the-art methods are presented in Table 3. As can be seen, the proposed approach achieved superior performance over the state-of-the-art methods in most tool categories. Table 4 lists the phase recognition results on the Cholec80 dataset using the ***CNN-SE-MSF-LSTM*** approach. The precision and recall of all phases and the mean values are presented. Additionally, a comparison with the leading methods is also presented in Table 5. The training times and inference times of the evaluated approaches are presented in Table 6.

**Figure 5:**
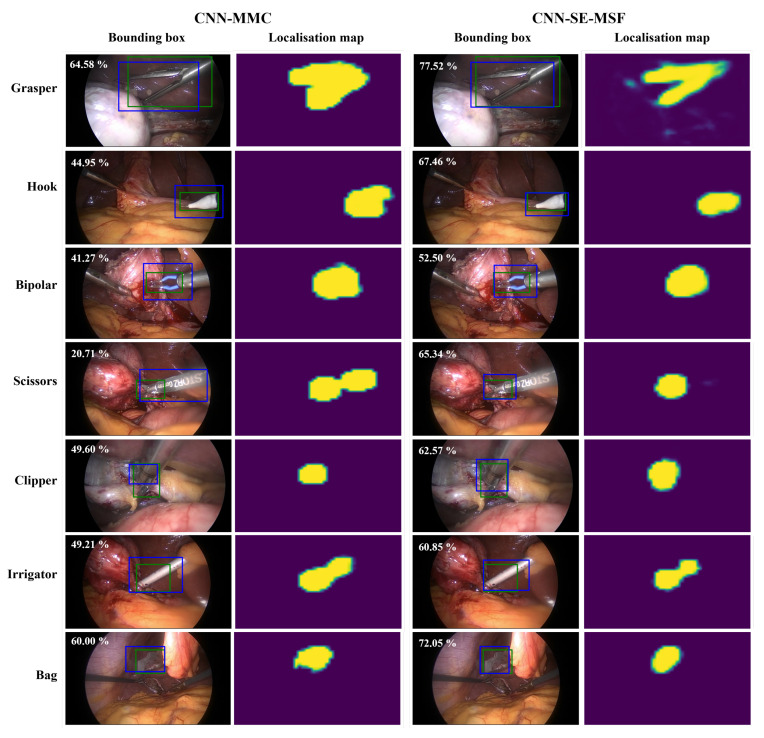
Visualization of localization maps of the CNN_MMC and CNN_SE_MF approaches for the seven surgical tools. For each tool, the manually labeled and predicted bounding boxes are visualized in green and blue, respectively. Images are labeled with the IoU (%) between the predicted and manually labeled boxes.

**Figure 6:**
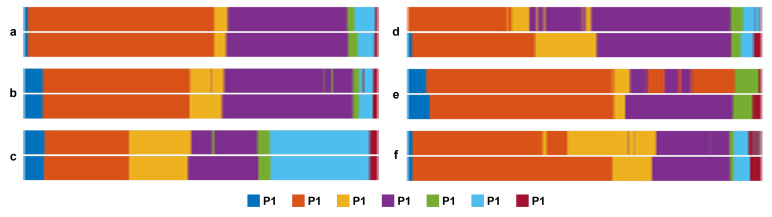
Color-coded visualization for phase recognition results of the top-3 (**a**,**d**,**c**) and bottom-3 (**d**,**e**,**f**) procedures. The ground truth is at the top, and the prediction is at the bottom.

In order to provide insight into the performance improvement achieved by the proposed approach, qualitative results for tool detection and phase recognition were visualized. Figure 5 visualizes localization maps of every tool obtained by the ***CNN-MMC*** and ***CNN-SE-MSF*** models. Every image contains the manually labeled bounding box and the predicted bounding box of the corresponding tool, and is labeled with the IoU value between the two boxes. The examined tool class probability was higher than 98% obtained by ***CNN-MMC*** or ***CNN-SE-MSF*** for all images. Figure 6 shows the predictions and ground truth of the top-3 and bottom-3 procedures for surgical phase recognition.

## 5. Discussion

This study presents a multi-task, weakly supervised deep learning approach trained by binary tool presence labels and phase labels to analyze laparoscopic videos. The approach is intended to recognize surgical phases and detect and discriminate between surgical tools. An extensive evaluation of the proposed model was conducted on the Cholec80 dataset [22].

### 5.1. Phase Recognition

The proposed approach yielded a mean recall and mean precision of 89.0% and 87.9%, respectively. These values improved on the base ResNet-50 model mean recall and precision values of 71.8% and 72.0%, respectively. Hence, the attention modules and combining features from multiple stages helped the model to leverage better phase-related feature representation of the laparoscopic image content. Additionally, the LSTM network contributed effectively to modeling the temporal constraints of surgical phases.

As can be seen from the precision and recall values in Table 4, the proposed approach achieved the best performance for P1, P2, and P4 with recall values of 94.6%, 95.8%, and 95.2%, respectively. Conversely, the results of other phases were lower, particularly for P6. This high variance between these phases is interpreted by the imbalanced data distribution, where P2 and P4 typically have longer periods than other phases in the cholecystectomy procedures. This can be seen from the mean duration of each phase presented in Table 1. The first four phases are performed consecutively (i.e., linear phase transitions), while the last three phases are associated with non-linear transitions. Consequently, P5, P6, and P7 had lower recognition performance. In a similar way, obtaining a high precision value of P2 (98.4%) interprets the high recognition results of P1 despite its low distribution in the dataset.

The tool-phase relation has already been addressed in other works and also described in the introduction and methodology sections. Therefore, it is worth noting that, there is a high correlation between the results obtained for tools and phases. For instance, the high hook presence detection performance of 99.7% matches the high recognition performances of P2 and P4, which are mainly performed with the hook tool. Furthermore, the improvements obtained by the ***CNN-SE-MSF-LSTM*** approach over the ***CNN-MMC*** for scissors explain the improvements obtained for P3 (Recall of 86.3%).

### 5.2. Tool Classification and Localisation

Experimental results show that adding the SE attention modules and combining features from low and high layers improved the tool classification performance over previous methods. Moreover, employing the LSTM network yielded the most notable improvement for all tools, particularly the scissors. ***CNN-SE-MSF-LSTM*** and ***CNN-SE-MSF*** achieved mAP values of 95.6% and 94.1%, respectively. These values exceeded the established ***CNN-MMC*** [42] mAP of 90.4% (see Figure 3) and imply the advantage of using attention modules and the MSF for tool classification. Moreover, modeling temporal dependencies along the video sequence helped refine classifications obtained by only employing spatial models.

Every surgical phase is performed by the surgeon using a specific set of tools. This explains the basis of developing a multi-task approach that jointly performs tool and phase recognition. Since the surgical phases are performed in a specific sequence, the tool’s appearance during the surgery is also somewhat constrained. Therefore, the best classification performance was achieved after employing the LSTM (Table 4). The AP of some tools (e.g., scissors) was enhanced by a larger margin over the spatial model, while other tools had a smaller improvement (e.g., grasper). This high variance in improvements between the tools can be interpreted by the surgical phases associated with these tools. For instance, the scissors were only required during the third phase (cutting and clipping) to cut the cystic duct. Hence, the LSTM learned discriminative temporal information for the scissors. On the other hand, the grasper was utilized during all phases to grasp tissues. Therefore, modeling temporal information provided negligible performance enhancement for the grasper classification.

Figure 4 shows the qualitative assessment of the ***CNN-MMC*** and ***CNN-SE-MSF-LSTM*** approaches. From the localization maps of each tool, it can be noticed that the ***CNN-SE-MSF*** was capable of learning the tool regions better than the base ***CNN-MMC*** model. The IoU values between the manually labeled and predicted bounding boxes show better localization performance using the ***CNN-SE-MSF*** approach. Moreover, adding the SE and MSF to the ***CNN-MMC*** helped smooth the localization map and make it look closer to the shape of the tooltip (Figure 5, grasper tool).

**Figure 7:**
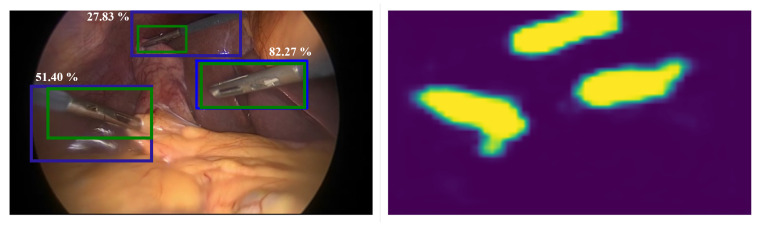
Multiple instances of the grasper (**left**) and the localization map (**right**). The green and blue boxes represent the manually labeled and predicted bounding boxes, respectively. Each bounding box is labeled by the IoU (%) value.

In the Cholec80 dataset, the grasper has multiple tool instances (i.e., up to three graspers may appear in the image) while all other surgical tools have a single tool instance. The proposed approach was designed to generate one localization map per tool, Nevertheless, multiple instances of the grasper were detected through a post-processing step. Here, the largest three objects in the localization map of the grasper were considered as ’detections’, and the bounding boxes were assigned the same confidence that represents the grasper presence probability. Figure 7 shows an example of multiple instances of the grasper and the detected bounding boxes. As can be seen, the proposed approach was able to localize the three instances of the grasper, however, only two of the ’detections’ were considered as $T_{P}$ with IoU greater than $T_{IoU}=0.5$. The region that contained the shaft of the third grasper (with IoU = 27.83%) was also detected in the localization map as part of the tool and not only the characteristic tip. Indeed, the weakly-supervised training of the proposed approach resulted in relatively larger bounding boxes that included the tip and parts of the tool shaft. In principle, the shaft is also part of the tool but according to the evaluation criteria of this work, only the tooltip should be localized. However, labeling the tool shaft with additional bounding boxes has the potential to better evaluate the performance of the model in terms of the ability to separate background information from tool regions, and potentially also capture the tool orientation as well as location.

**Figure 8:**
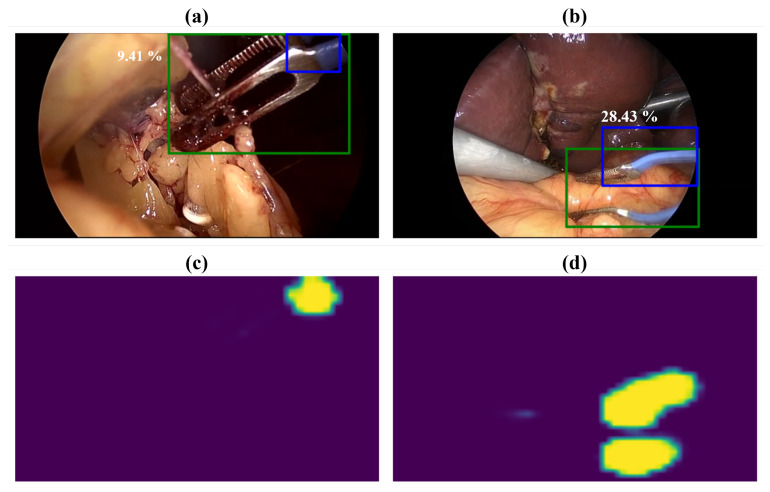
Examples of false detections of the bipolar bounding box. (**a**,**b**) show laparoscopic images of the bipolar with manually labeled (green) and predicted (blue) bounding boxes. Each bounding box is labeled by the IoU (%) value; (**c**,**d**) represent the localization maps obtained by the proposed approach for the bipolar.

Figure 8 shows that the proposed approach failed to detect the bounding box precisely, even in cases when the activated regions matched the tool location in the image. For the first image (Figure 8a), the manually labeled and predicted bounding boxes of the bipolar are presented in green and blue, respectively. The feature map obtained for the bipolar is also shown in Figure 8c. The bipolar partially appears in the image, and only a small part of the tip was detected. The bipolar tip consists of two parts, a characteristic blue clevis part and the grasping part that has a similar appearance to the grasper tip. Therefore, the detection of the bipolar relied on localizing its blue clevis, not the entire tooltip. Both the tooltip and the clevis was considered for labeling the Cholec80-Boxes. Hence, the labeling protocol can be modified accordingly by only considering the characteristic clevis of the bipolar. Similarly, the clevis of the bipolar in Figure 8b was detected by the proposed approach as two separate objects as can be seen in the localization map of the bipolar (Figure 8d). In the post-processing step, only one object was counted for detecting the bipolar bounding box, which lead to a false prediction with IoU = 28.43%. Therefore, the rate of this kind of false detection could potentially be ameliorated by refining the post-processing step (e.g., morphological image processing).

### 5.3. Comparison with The State-of-the-Art

Table 3 and Table 5 present comparisons with the leading methods for surgical tool presence detection and phase recognition. Twinanda et al. introduced the base model EndoNet that performed tool presence detection and phase recognition in a multi-task manner [22]. HHMM was employed to model temporal dependencies between surgical phases. They achieved a mAP of 81.0% for tool presence detection and a mean precision and mean recall of 73.7% and 79.6% for surgical phase recognition, respectively. Jin et al. tackled tool detection and phase recognition tasks in a similar fashion but employed an LSTM network as a temporal model. They also introduced a novel loss function that better considered the tool-phase relation. Their methods showed great improvements over the EndoNet with a mAP of 89.1% for tool detection and a mean recall of 88% for phase recognition. However, in both approaches, tool presence detection was carried out solely based on spatial information learned by the CNN model. On the contrary, this work proposed using the LSTM network for both phase recognition and tool presence detection tasks.

Wang et al. [38] proposed using a graph convolutional neural network (GCN) to learn temporal information from short video clips for the tool classification task. They evaluated their methodology on the Cholec80 dataset and reported a value of 90.1% for mAP. In a recent study [35], two stages of temporal modeling were proposed to learn dependencies, first from short video sequences of unlabeled frames and then across the whole surgical video. This approach yielded the best performance results reaching mAP of 94.6% between other tool presence detection methods [35]. Vardazaryan et al. [42] transferred the weakly-supervised WILDCAT approach [21] into the tool localization in laparoscopic videos. Indeed, the ***CNN-MMC*** (Table 2) approach represents a reproduction of their work. Nwoye et al. [39] built upon work in [42] and employed a convolutional LSTM layer to learn spatiotemporal coherence along the video sequence. Similar to this study, both approaches Vardazaryan et al. [42] and Nwoye et al. [39] were trained only with the binary tool labels, and they reported the tool presence of mAP at 87.2% and 92.9%. Interestingly, the model presented in this study (***CNN-SE-MSF-LSTM***) achieved higher mAP values than [35,38,39,42] at 95.6%. The tool localization results of this were not compared with other works because of different types of evaluation data.

For phase recognition, Jin et al. [29] proposed the SV-RCNet deep learning approach, which is composed of a CNN and LSTM network. They also introduced a prior knowledge inference scheme. Their method yielded a high recognition performance with mean precision and mean recall of 88.1% and 88.9%, respectively. Recently, Czempiel et al. [30] proposed using a temporal convolutional network, and they reported 80.9% and 87.4% precision and recall, respectively. In [52], combining a CNN with a two-stage LSTM, the authors achieved 92.9% accuracy on the Cholec80. Recent studies proposed using transformers instead of LSTM networks for temporal modeling. Czempiel et al. [32] proposed the OperA approach based on a transformer model. They reported 82.2% and 86.9% for precision and recall, respectively. Indeed, the ***CNN-SE-MSF-LSTM*** exceeded the performances of most state-of-the-art methods for phase recognition (Table 5) and achieved the best recall value of 89.0% and 88.5% precision.

### 5.4. Limitations and Future Scope

An experimental evaluation of the proposed approach was carried out using a single dataset (Cholec80). To justify the robustness and generalization capability of this approach, extensive evaluations with other datasets should be performed. Furthermore, the spatial and temporal models were trained separately, not end-to-end. Indeed, end-to-end training is the main drawback related to its computational burden, where a large GPU memory is required. Nevertheless, end-to-end training can be done using short image sequences to leverage better spatial–temporal features. The LSTM can then be trained with complete video sequences.

The developed framework has the potential to be employed as a first step in labeling new datasets. For instance, bounding boxes can be generated and then modified by labeling specialists. Consequently, manual tagging to support DL model development could be achieved with less time and effort.

## 6. Conclusions

This study proposed a deep learning approach for surgical phase recognition, tool presence detection, and weakly-supervised tool localization. A CNN-based model was modified by adding SE attention modules and fusing features from multiple stages to enable a better representation of the image input space. Temporal information was also modeled using an LSTM network. The thorough quantitative evaluation and qualitative analysis of the proposed approach demonstrated high tool presence detection performance that exceeded all state-of-the-art methods. Furthermore, the phase recognition performance was comparable to previous studies and had the best sensitivity among them. Finally, the tool localization performance achieved showed that this approach has the potential to be integrated into intelligent systems that require automatic localization of surgical tools.

## Figures and Tables

**Table 1:** Surgical phases and tools in the Cholec80 dataset; √ indicates the phases in which the tool is often used.

Number	Phase	Duration Mean ± Std.	Grasper	Bipolar	Hook	Scissors	Clipper	Irrigator	Specimen Bag
P1	Preparation	107±103	√	x	x	x	x	x	x
P2	Calot Triangle Dissection	935±663	√	√	√	x	x	x	x
P3	Clipping and Cutting	176±128	√	x	x	√	√	x	x
P4	Gallbladder Dissection	730±533	√	√	√	x	x	√	x
P5	Gallbladder Packaging	95±48	√	x	x	x	x	x	√
P6	Cleaning and Coagulation	179±156	√	√	x	x	x	√	√
P7	Gallbladder Retraction	83±75	√	x	x	x	x	x	√

**Table 2:** Description of the evaluated approaches.

Approach	Description
***CNN-MMC***	The CNN model combined with the MMC layer and tool-wise and spatial pooling.
***CNN-SE-MSF***	The CNN-MMC approach but adapted by adding SE attention modules and the MSF.
***CNN-SE-MSF-LSTM***	The CNN-SE-MSF was combined with an LSTM network to model temporal dependencies along the video sequence.

**Table 3:** Comparison of surgical tool presence detection results (%) of different approaches on the Cholec80 dataset (the best performances are indicated in bold).

Tool	EndoNet [22]	MTRCNet [27]	Nwoye [39]	GCN [38]	ResNet-LC-LV [35]	*CNN-SE-MSF-LSTM*
Grasper	84.8	84.7	**99.7**	-	87.4	91.0
Bipolar	86.9	90.1	95.6	-	95.9	**97.3**
Hook	95.6	95.6	99.8	-	99.4	**99.8**
Scissors	58.6	86.7	86.9	-	**92.7**	90.3
Clipper	80.1	89.8	97.5	-	**98.5**	97.4
Irrigator	74.4	88.2	74.7	-	91.4	**95.6**
Specimen bag	86.8	88.9	96.1	-	96.6	**98.3**
Mean	81.02	89.1	92.9	90.1	94.6	**95.6**

**Table 4:** Precision and recall of phase recognition results using the proposed approach on the Cholec80 dataset.

Phase	Precision		Recall	
	ResNet-50	*CNN-SE-MSF-LSTM*	ResNet-50	*CNN-SE-MSF-LSTM*
P1	71.8	98.0	54.3	94.6
P2	84.0	98.4	85.1	95.8
P3	73.7	80.8	69.7	86.3
P4	85.4	92.4	84.7	95.2
P5	62.5	80.1	79.0	87.6
P6	68.7	83.5	71.2	75.9
P7	58.2	86.3	58.4	84.7
Mean	72.0	88.5	71.8	89.0

**Table 5:** Comparison of phase recognition results (%) of different approaches on the Cholec80 dataset (best performances are indicated in bold).

Approach	Accuracy	Precision	Recall
EndoNet [22]	81.7	73.7	79.6
SV-RCNet [29]	90.7	88.1	88.9
MTRCNet [27]	89.2	86.9	88.0
TeCNO [30]	89.0	80.9	87.4
OperA [32]	91.2	82.2	86.9
Jalal et al. [52]	92.9	**90.1**	85.1
***CNN-SE-MSF-LSTM***	**93.1**	88.5	**89.0**

**Table 6:** Computation times of the evaluated approaches. An NVIDIA RTX A6000 GPU was used for implementation.

Approach	Training (h)	Test (ms/image)
CNN-MMC	17.5	20
CNN-SE-MSF	20	24
CNN-SE-MSF-LSTM	30	25

## Data Availability

The data presented in this study were composed of two datasets (Cholec80 and Cholec80-Boxes). The Cholec80 dataset is available (http://camma.u-strasbg.fr/datasets/ (accessed on 22 March 2017)) from the publisher upon request. The Cholec80-Boxes dataset is available from the corresponding author upon reasonable request.

## Abbreviations

The following abbreviations are used in this manuscript:

OR	operating room
CAS	context-aware system
SDS	surgical data science
DL	deep learning
CNN	convolutional neural network
HMM	hidden Markov model
LSTM	long short-term memory
NARX	nonlinear autoregressive network with exogenous input
MS-TCN	multi-stage temporal convolutional network
GCN	graph convolutional network(s)
GAN	generative adversarial network
Grad-CAM	gradient class activation map
ReLU	rectified linear unit
FC	fully-connected
SE	squeeze-and-excitation
GAP	global average pooling
MSF	multi-stage feature fusion
MMC	multi-map convolutional layer
mAP	mean average precision
IoU	intersection over union
PR	precision
RE	recall
